# LncRNA Functions as a New Emerging Epigenetic Factor in Determining the Fate of Stem Cells

**DOI:** 10.3389/fgene.2020.00277

**Published:** 2020-03-31

**Authors:** Jingcheng Chen, Yizhuo Wang, Cong Wang, Ji-Fan Hu, Wei Li

**Affiliations:** ^1^Cancer Center, The First Hospital of Jilin University, Changchun, China; ^2^VA Palo Alto Health Care System, Stanford Medical School, Stanford University, Palo Alto, CA, United States

**Keywords:** long non-coding RNA, stem cell, pluripotency, cell differentiation, reprograming, epigenetics, promoter-interacting lncRNA network

## Abstract

Pluripotent stem cells have broad applications in regenerative medicine and offer ideal models for understanding the biological process of embryonic development and specific diseases. Studies suggest that the self-renewal and multi-lineage differentiation of stem cells are regulated by a complex network consisting of transcription factors, chromatin regulators, signaling factors, and non-coding RNAs. It is of great interest to identify RNA regulatory factors that determine the fate of stem cells. Long non-coding RNA (lncRNA), a class of non-coding RNAs with more than 200 bp in length, has been shown to act as essential epigenetic regulators of stem cell pluripotency and specific lineage commitment. In this review, we focus on recent research progress related to the function and epigenetic mechanisms of lncRNA in determining the fate of stem cells, particularly pluripotency maintenance and lineage-specific differentiation. We discuss the role of the *Oct4* and *Sox2* promoter-interacting lncRNA as identified by Chromatin RNA *In Situ* reverse Transcription sequencing (CRIST-seq). Further understanding of their potential actions will provide a basis for the development of regenerative medicine for clinical application. This work offers comprehensive details and better understanding of the role of lncRNA in determining the fate of stem cells and paves the way for clinical stem cell applications.

## Introduction

Pluripotent stem cells (PSCs), including ESCs and iPSCs, have great potential in regenerative medicine. ESCs are derived from the ICM of a mammalian blastocyst and can differentiate into different cell types to form the endoderm, mesoderm, and ectoderm (Thomson et al., 1998). On the other hand, iPSCs are induced by pluripotent reprograming of somatic cells with specific transcription factors (Takahashi and Yamanaka, 2006; Yu et al., 2007). PSCs have the ability to self-renew and differentiate into different cell types, making them ideal models for exploring embryonic development early events and understanding regenerative medicine (Borsos and Torres-Padilla, 2016). It is well known that the function of PSCs is regulated by many growth factors, including LIF, BMPs, FGF4, and their receptor-related signaling (Chen C.Y. et al., 2017; Hassani et al., 2019). PSCs express characteristic transcription factors, such as *Oct4*, *Nanog*, *Sox2*, and *Klf4*. The collaboration and synergy of these transcription factors are crucial for maintaining stemness and pluripotency. The cell fate of PSCs is controlled or regulated by a complex process, involving transcription factors, signaling pathways, non-coding RNAs and their collaborative interaction. Hence, understanding the molecular mechanisms underlying determination of PSC fate will be of significance in manipulating their self-renewal and differentiation into specific cell lineages for potential application.

Long non-coding RNA refers to a family of non-coding RNAs with more than 200 bp in length and is transcribed by RNA polymerase II, usually 5′ capped, spliced and polyadenylated (Kung et al., 2013; Quinn and Chang, 2016). LncRNA can be transcribed from different genome regions, including introns, exons, intergenic connections, and other areas. Currently, there are about 30000 lncRNAs identified in human and mice (Frankish et al., 2019), but only a handful part of them have been recognized for their function. Structurally, lncRNA is less evolutionally conserved than mRNA (Ponting et al., 2009), and they can display complicated secondary architecture after interacting with proteins, DNA or RNA to form a proper tertiary structure for their functional activities (Wang and Chang, 2011). LncRNA acts as essential regulators in a variety of biological or cellular processes, including chromatin remodeling, transcription, post-transcriptional processing, intracellular trafficking, metabolism, development and differentiation (Ponting et al., 2009; Wapinski and Chang, 2011). LncRNA has the ability to regulate the self-renewal and differentiation of PSCs (Mercer et al., 2009; Cao, 2014). Here, we provide an updated overview on how lncRNA epigenetically regulates PSC function.

## The Role of LncRNA in Stem Cell Pluripotency

Stem cell pluripotency and differentiation are regulated by a multilayered and complicated network, including lncRNA. Since PSCs hold therapeutic potential in many types of diseases, understanding the regulatory network for maintenance of stem cell pluripotency will be essential for manipulating PSCs in clinical applications. Previous studies have demonstrated that lncRNAs are crucial regulators for regulating stem cell pluripotency (Chen and Zhang, 2016; Rosa and Ballarino, 2016). Transcriptional analysis of human fibroblasts, their derivative iPSCs, and ESCs showed that 133 lncRNAs were up-regulated and 104 lncRNAs were down-regulated in iPSCs and ESCs as compared with fibroblasts (Loewer et al., 2010). The upregulated lncRNAs may bind to *Oct4, Sox2* and *Nanog* in iPSCs, indicating that they are crucial for maintenance of stem cell pluripotency. In a second study, more than 130 lncRNAs were examined by shRNA knockdown for their function in the of ESC pluripotency maintenance (Guttman et al., 2011). ChIP-Seq was performed to generate a database to illustrate the relationship between transcription factors and lncRNAs in pluripotency regulation (Yang et al., 2013). Most lncRNAs were regulated by key pluripotent transcription factors, including *Oct4, Sox2* and *Nanog* (Ng et al., 2012; Fico et al., 2019). Recently, Du and coauthors identified multiple pluripotency-associated lncRNAs embedded in the chromatin regulatory network by combining RNA-Seq and RAT-seq (Du et al., 2018).

**FIGURE 1:**
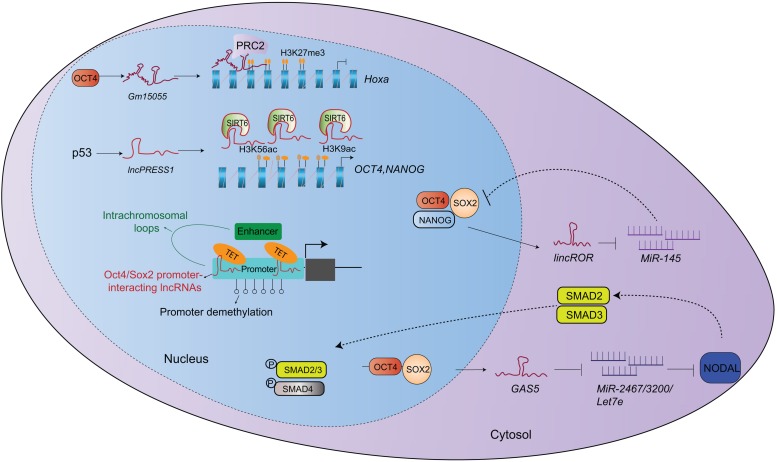
Mechanisms of lncRNA in regulating stem cell pluripotency. Nuclear lncRNAs regulate the expression of pluripotent genes via chromatin remodeling. While lncRNAs in cytosol mainly act as post-transcriptional regulators. They work as miRNA sponges to affect gene expression or signaling pathway.

The intracellular localization of a lncRNA provides a clue for its action mode (Batista and Chang, 2013). Nuclear lncRNAs often interact with chromatin modification factors, RNA binding proteins or transcription factors to regulate gene expression (Figure 1). The lncRNA *Gm15055* is highly induced by *Oct4* and is located near the *HOX* gene cluster of mESCs. *Gm15055* can interact with and recruit PRC2 to maintain H3K27me3 levels on *HoxA* genes (Liu et al., 2016). The expression of lncRNA p53-regulated and ESC-associated 1 (*LncPRESS1*) is suppressed by p53 (Jain et al., 2016), and its knockdown induces spontaneous differentiation in hESCs by down-regulating *OCT4*, *NANOG* and *c-MYC*, but it up-regulates *HOXA2*, *HOXB1*, and *FOXA2* expression. Mechanistically, *lncPRESS1* can interact with SIRT6, inhibiting SIRT6 attachment to chromatin and maintaining the histone H3K56 and H3K9 promoter acetylation to safeguard hESC pluripotency (Jain et al., 2016). *LncKdm2b*, a *Kdm2b* divergent lncRNA, is a positive regulator of ESC self-renewal and early embryogenesis. It recruits Snf2-related CREBBP activator protein (SRCAP)-contained remodeling complex to the *Zbtb3* promoter and activates *Zbtb3* expression to induce *Nanog* expression (Ye et al., 2018). Another study reports that *lncRNA-ES1* (AK056826), *lncRNA-ES2* (EF565083) and *lncRNA-ES3* (BC026300) are differentially expressed in hESCs nuclei, and they can bind near the TSS of the *OCT4* and *NANOG* promoters to enhance hESC pluripotency (Ng et al., 2012). Their deficiency reduces the pluripotency-related gene expression, but enhances the neuroectoderm, endoderm and mesoderm germ layer-related gene expression. *LncRNA-ES1* and *lncRNA-ES2* physically interact with SUZ12 and SOX2, and they act as modular scaffolds for SUZ12 (or PRC2) and SOX2 in hESCs. SOX2 may induce H3K27 methylation during the neuroectoderm lineage commitment, leading to gene silencing at these loci. Furthermore, SOX2 binds lncRNAs to prevent the binding of other core pluripotency-associated transcription factors. LncRNA-ES3 silencing displays a phenotype similar to *NANOG* knockdown in hESCs (Ng et al., 2012). *Zeb2-NAT* is a *Zeb2* antisense lncRNA that can be upregulated in fibroblasts with aging. *Zeb2-NAT* deficiency increases the reprograming efficiency of aged fibroblasts into iPSCs and maintains ESC self-renewal and pluripotency. Hence, *Zeb2-NAT* is an early marker for pluripotency loss (Bernardes de Jesus et al., 2018).

Cytoplasmic lncRNAs are functionally different from nuclear lncRNAs, and mainly act as post-transcriptional regulators by modulating mRNA degradation and protein translation (Figure 1). By comparing ESCs and fibroblasts, Loewer et al. identified 28 “iPSC-enriched” lncRNAs. Among them, *linc-RoR* regulated reprograming efficiency by binding to OCT4, *SOX2*, and *NANOG* (Loewer et al., 2010). Further study revealed that *linc-RoR* acted as a ceRNA and sponges *miRNA-145* to enhance *OCT4*, *SOX2*, and *NANOG* expression and self-renewal in iPSCs (Wang et al., 2013). The lncRNA *GAS5*, a tumor suppressor, is highly expressed in hESC cytoplasm and is induced by OCT4 and SOX2. Knockdown of *GAS5* results in stem cell differentiation by reducing hESCs in the G2/M phase, indicating that *GAS5* promotes hESC self-renewal cells. *GAS5* can sponge *miR-2467-5p*, *miR-3200-3p* and *let-7a/e-5p* to enhance the NODAL signaling (Xu et al., 2016). The *Cyrano* lncRNA is located in ESC cytoplasm and nuclei. It directly interacts with *miR-7* to enhance *Nanog* expression and ESC self-renewal (Smith et al., 2017). *Cyrano* silencing inhibits ESC self-renewal and survival, and it downregulates *Nanog* expression (Smith et al., 2017). The *lincU* lncRNA is highly expressed in the cytoplasm of naïve state stem cells in a *Nanog*-dependent manner. *LincU* can stabilize Dusp9 from ubiquitination proteasome-mediated degradation to inhibit the MAPK/ERK signaling, but it enhances mESC pluripotency (Jiapaer et al., 2018).

Collectively, although lncRNAs regulate the pluripotency by varying mechanisms, they center on the stemness-related *Oct4, Sox2* and *Nanog* expression and protein stabilization in PSCs. Regulation of these transcription factors is crucial for self-renewal of different PSC types.

## LncRNA Acts as a Novel Class of Chromatin RNA Regulatory Factor in Pluripotent Reprograming

Terminally differentiated somatic cells can be reprogramed to pluripotent status as iPSCs by ectopic expression of defined factors. However, this reprograming process is time-consuming and extremely inefficient, which hinders potential clinical applications for regenerative medicine. To explore the mechanisms underlying reprograming, Zhang and coauthors used the chromatin conformation capture (3C) approach to compare promoter DNA binding and chromatin architecture between iPSCs and the “non-iPSCs” that expressed lentiviral reprograming-initiating factors, but failed to complete reprograming to pluripotency. It is interesting to note that the formation of a promoter-enhancer intrachromosomal loop architecture is a critical epigenetic barrier that must be overcome for the successful pluripotency induction (Zhang et al., 2013; Hu and Hoffman, 2014). The non-iPSCs could not achieve pluripotency, partially due to the lack of this intrachromosomal looping (Zhang et al., 2013; Hu and Hoffman, 2014). Thus, it is critical to identify molecular factors that orchestrate this pluripotency-specific intrachromosomal loop.

**FIGURE 2:**
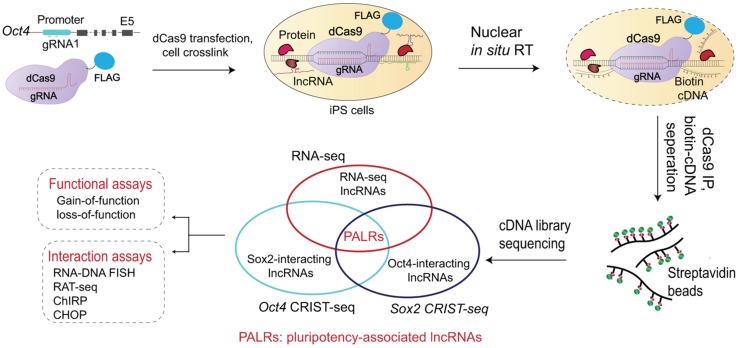
Mapping stemness gene promoter-interacting lncRNA by CRIST-seq. The CRIST-seq assay combines the specificity of Cas9 gene targeting with the simplicity of biotin-lncRNA labeling. Target cells are first transfected with lentiviruses carrying dCas9-FLAG/gRNA that target the promoter of stemness genes, like *Oct4* and *Sox2*, that are two core stem cell factors essential for the establishment and maintenance of pluripotency. The cells are crosslinked to fix the promoter chromatin DNA-lncRNA complex. Following lysis of the cell membrane, the promoter-interacting RNAs are reverse transcribed *in situ* into biotin-cDNAs (cDNAs) in the isolated nuclei with biotin-dCTP. The promoter chromatin-cDNA complex is immunoprecipitated by a Cas9-FLAG antibody and the promoter-interacting biotin-cDNAs are separated by the biotin-streptavidin bead purification. The CRIST-captured cDNAs are used for library construction and Illumina sequencing is performed for identifying the lncRNAs that interact with the promoter of stemness genes. Integration of the *Oct4-Sox2* CRIST-seq data with the RNA-seq data allows the identification of lncRNAs that not only interact with core factor promoters of stem cells, but are also differentially expressed during reprograming. The lncRNA-promoter interaction is subsequently validated by other tools, including RNA-DNA FISH, RAT, ChIRP, and CHOP assays. Gain- and loss-of-function assays are then used to characterize the role of identified lncRNAs in stem cells. The CRIST-seq method offers a more efficient strategy for exploring the lncRNA network in the regulation of reprograming and pluripotency.

Long non-coding RNA consists of essential components of the three-dimensional genome structure. Several previous studies have identified lncRNAs that mediate the formation gene regulatory chromosome loops (Zhang et al., 2014; Pisignano et al., 2019). Functionally, these chromatin loop structures may bring distant enhancer elements close to the core promoter for optimal gene expression (Zhang et al., 2013; Wang H. et al., 2015). Recently, Zhang et al. (2019) reported to the CRIST-seq approach to map lncRNA within regulatory elements of stemness genes (Figure 2). They profiled the lncRNA interaction network in the *Sox2* and *Oct4* promoters using this approach. Among the identified lncRNAs, *Snhg14* was abundantly expressed in iPSCs and ESCs. Functional assays confirmed that *Snhg14* was required to maintain stem cell pluripotency. Jia et al. (2020) later used CRIST-seq/RNA-seq to identify *Oplr16* (*Oct4* promoter-interacting LncRNA 16) as another pluripotency-associated chromatin RNA factor. *Oplr16* enhanced reprograming epigenetically by coordinating intrachromosomal looping and DNA methylation in the *Oct4* promoter. Wang et al. (2020) used CRIST-seq to identify *Peblr20* (*Pou5F1* enhancer binding lncRNA 20) as another reprograming-associated lncRNA. Notably, *Peblr20* binds to the *Oct4* enhancers, where it recruits TET2 demethylase and activates the enhancer RNA (eRNA) pathway. Thus, *Peblr20* functions as a critical pluripotent lncRNA that utilizes a novel *trans* epigenetic eRNA mechanism to control stem cell fate. Exploration of the genome-wide binding sites of central transcription factors and the lncRNA interactome during cell fate conversion may promote comprehensive understanding of the transcription network in reprograming.

## LncRNA as a Promising Epigenetic Regulator of Stem Cell Differentiation and Specific Cell Lineage Commitment

Pluripotent stem cells can differentiate and commit to a specific lineage in a given environment. The acquisition of cellular identities is a well-organized, precisely timed, and robustly executed process. The lack of effective differentiation and accurate specification of cell types hindered the widespread use of stem cells. Lineage differentiation of stem cells is regulated by a diverse and complex system that includes lncRNA. We will discuss the role of lncRNA in cell lineage commitment and focus on how lncRNA regulates PSC differentiation into adipocytes, osteogenic, muscle, cardiomyocytes, neurons, skin, and blood cells.

## LncRna in the Adipogenic Differentiation of Stem Cells

Long non-coding RNA is involved in the adipogenic differentiation of stem cells. Understanding the mechanisms underlying adipogenesis regulation by lncRNA may reveal new therapeutic targets to combat obesity and its related diseases (Tye et al., 2015).

Long non-coding RNA *SRA* is the first lncRNA identified for functional adipogenesis promotion (Lanz et al., 2003). *SRA* can enhance the expression of PPARγ, a main adipogenesis regulator. *SRA* silencing inhibits the differentiation of pre-adipocytes 3T3-L1 cells. Furthermore, *SRA* can enhance insulin sensitivity by promoting cell cycling, inhibiting inflammatory gene expression and the TNFa-induced phosphorylation of c-Jun NH2-termianl kinase in pre-adipocytes. *SRA*^–/–^ mice are lean with reduced adipogenic gene expression (Sheng et al., 2018). The lncRNA adipogenic differentiation-induced non-coding RNA (*ADINR*) is induced by adipogenesis and positively regulates both PPARγ and CEBPA expression by recruiting PA1 (a component of the MLL3/4 complex) to the *CEBPA* promoter and impacting H3K4me3 and H3K27me3 histone modification (Xiao et al., 2015). In addition, lncRNA *Blnc1* can promote adipocyte differentiation with essential transcription factors Ucp1, PPARγ and Ebf2. Functionally, *Blnc1* can interact with and recruit Ebf2 to enhance Ucp1 expression and mitochondrial respiration-associated adipogenic differentiation (Zhao et al., 2014).

Maternally expressed gene 3 was identified as an osteogenic differentiation-related lncRNA in BMSCs (Zhuang et al., 2015). A recent study has shown that *MEG3* can inhibit adipogenic differentiation by sponging miR-140-5p because *MEG3* silencing enhances adipogenic differentiation in pre-adipocytes (Li Z. et al., 2017). It is well known that miR-140-5p can enhance adipogenic differentiation by upregulating PPARγ and CEBPA expression, two principal adipogenic transcription factors (Li Z. et al., 2017). Furthermore, *ADNCR* is an inhibitory lncRNA of adipogenic differentiation that functions as a ceRNA for *miR-204* to promote the miR-204-targeted SIRT1 expression, given that SIRT1 can inhibit adipocyte differentiation by interacting with NCoR and SMART to suppress PPARγ activity. Hence, *ADNCR* can enhance the adipogenesis inhibitory SIRT1 expression in a miR-204-dependent manner (Li et al., 2016). Thus, lncRNA can regulate adipogenesis by directly interacting with chromatin modification complexes or transcription factors. LncRNA can also act as ceRNAs for miRNAs to influence adipogenesis regulator expression. These lncRNAs form a network to regulate the dynamic adipogenesis process. However, it is still unclear how these lncRNAs interact to maintain adipogenesis homeostasis.

## The Role of LncRNA in Osteogenic Differentiation

Bone mesenchymal stem cells can differentiate into osteoblasts, chrondrocytes, and osteocytes in osteogenic differentiation for bone tissue formation. Osteogenic differentiation stimulates ALP expression and calcium deposition. Osteogenic differentiation is stimulated and regulated by genetic and environmental factors, including lncRNA. Its deficiency can cause osteogenesis imperfecta in young children and osteoporosis, a common disease affecting many people, particularly for the elderly people and postmenopausal women in the world. Hence, understanding lncRNA regulatory roles in osteogenesis may reveal potential therapeutic targets for intervention of osteogenesis deficient diseases (Long, 2011).

Long non-coding RNA can act as miRNA spongers to modulate the miRNA-targeted gene expression (Salmena et al., 2011). Human BMSC osteogenesis induce *H19* and *linc-ROR* lncRNAs. *H19* can upregulate osteogenic related gene expression and promote bone formation *in vivo* by targeting *miR-141* and *miR-22*, two potent inhibitors of osteogenesis. These miRNAs can down-regulate β-catenin expression, attenuating the Wnt/β-catenin signaling for osteoblast development. The action of *H19* is counteracted by its encoded *miR-675-5p* that inhibits osteoblast differentiation (Liang et al., 2016). Accordingly, *H19* can enhance osteogenesis by targeting *miR-141* and *miR-22* to avoid their inhibition of the Wnt/β-catenin signaling. Similarly, *linc-ROR* can also enhance the expression of osteogenic genes by sponging *miR-138* and *miR-145* to enhance ZEB2 expression and downstream Wnt/b-catenin signaling eventually (Feng L. et al., 2018). LncRNA-*MEG3* and lncRNA-*AK141205* can enhance osteogenesis by dissociating SOX2 from the *BMP4* promoter to induce BMP4 expression and by positively promoting CXCL13 expression, respectively (Zhuang et al., 2015).

On the other hand, osteogenic differentiation of hASCs down-regulates lncRNA *MIAT* expression. This, together with the fact that *MIAT* deficiency promotes osteogenic differentiation *in vitro* and accelerates bone formation *in vivo*, indicates that *MIAT* inhibits osteogenic differentiation. *MIAT* silencing reverses the TNFa-inhibited osteogenic differentiation. Mechanistically, *MIAT* can sponge *miR-150-5p* and interact with AKT to decrease oxidative stress and inflammatory factor stimulation (Shen et al., 2016; Jin et al., 2017). Similarly, osteogenic differentiation of HBMSCs down-regulates the expression of lncRNA *DANCR*, indicating that *DANCR* is an inhibitor. Indeed, *DANCR* deficiency elevates ALP and osteogenic marker gene expression, enhances cell cycling in the S phase, and *DANCR* over-expression has opposite effects (Zhang et al., 2018). Mechanistically, *DANCR* inhibits HBMSC osteogenic differentiation by targeting the p38 MAPK signaling, which can promote HBMSC differentiation, mineralization and proliferation (Zhang et al., 2018). In addition, osteogenic differentiation of BMSCs can also down-regulate lncRNA brain-derived neurotrophic factor-antisense transcript (*BDNF-AS*) and *MIR31HG* expression. *BDNF-AS* is transcribed from the *BDNF* antisense, while *MIR31HG* is from chromosome 9 and can be induced by the activated NF-kB. *BDNF-AS* can inhibit osteogenesis possibly via inverse regulation on *BDNF* and other osteogenic signaling (Feng X. et al., 2018). *MIR31HG* downregulation dramatically promotes osteogenic differentiation and significantly overcomes the osteogenesis inhibition induced by hASC inflammation. *MIR31HG* can interact with the NF-κB to inhibit bone formation by binding to IκBa to insult IκBa phosphorylation and subsequent NF-κB activation in hASC. Hence, *MIR31HG* and NF-κB form a regulatory loop to improve osteogenesis efficiency in hASCs under inflammatory microenvironment. *MIR31HG* may be a therapeutic target for inhibiting inflammation and enhancing bone formation (Jin et al., 2016). Moreover, lncRNA *ANCR* is crucial for maintaining the undifferentiated cell state in human epidermis. A recent study reveals that *ANCR* silencing promotes osteoblast differentiation because *ANCR* can directly interact with EZH2 to catalyze H3K27me3 in the *Runx2* promoter, inhibiting *Runx2* expression (Zhu and Xu, 2013).

## LncRNA in Myogenesis

Myogenesis is a highly ordered process that can be divided into several steps, including muscle stem cell activation, myoblast proliferation and differentiation, and myotubular formation. The process occurs in both postnatal development and the skeletal muscular regeneration after injury. It is orchestrated by a complex network involving in epigenetic regulators, transcription factors and lncRNA.

*Linc-MD1*, a muscle-specific lncRNA, is expressed in the cytoplasm of skeletal muscle cells and activated during myoblast differentiation. *Linc-MD1* can promote myogenesis by sponging *miR-133* and *miR-135* to enhance the expression of MAML1 and MEF2C, which are critical transcription factors to induce muscle-specific gene expression (Cesana et al., 2011). During the embryoid development, lncRNA *H19* expression is up-regulated in maternal embryonic tissues, but it down-regulated after birth only in the skeletal muscles. *H19* expression is strongly induced during myoblast differentiation while H19 deficiency inhibits skeletal muscule differentiation. *H19* exon1 encodes *miR-675-3p* and *miR-675-5p*, which can be induced during skeletal muscule differentiation. These two microRNAs can target the transcription factor Smad to impair BMP signaling and Cdc6 expression (Dey et al., 2014b). Besides, *H19* can also enhance Igf2 expression, a myogenesis stimulator in myoblasts (Kallen et al., 2013) and block the SIRT1/FoxO1 signaling (Xu et al., 2017). *Dum*, is induced by MyoD when myoblast differentiation begins and exclusively expressed in active myogenesis, but not in mature muscle homeostasis. *Dum* silencing dramatically delays myogenic differentiation. Mechanistically, the *Dum* gene is near *Dppa2*, a positive regulator of the pluripotency factor Oct4. *Dum* can *cis*-promote myogenesis to inhibit *Dppa2* expression by interacting and recruiting Dnmt to CpG islands in the *Dppa2* promoter (Wang L. et al., 2015). *MyoD* is crucial for myoblasts development and function. *MyoD* controls early myogenesis by inducing myoblast cell cycle arrest and initiating its differentiation program. Interestingly, the *LncMyoD* gene is located at about 30 kb upstream of the mouse *MyoD* gene and is specifically expressed in myoblasts during early differentiation. *LncMyoD* deficiency significantly inhibits terminal myoblasts differentiation due to continual cell cycling. *LncMyoD* is a *MyoD* target that can directly bind to the protein IMP2, and compete for IMP binding to inhibit cell cycling and enhance differentiation (Gong et al., 2015). Another lncRNA *MUNC* is transcribed from about 5 kb upstream of the *MyoD* gene. *MUNC* works as a DRR-enhancer RNA to activate myogenic gene expression (Mueller et al., 2015). Overall, lncRNAs are important regulators for skeletal muscle development.

A set of myogenesis-associated lncRNAs, *Yam 1-4*, can interact with YY1 and regulate myogenic differentiation (Lu L. et al., 2013). The *Yam-1* gene is located on chromosome 17 and can bind the 687 bp upstream of the *YY1* promoter. It is expressed in the nucleus and cytoplasm of myogenic progenitors (Lu L. et al., 2013). *Yam-1* can inhibit myogenic differentiation because *Yam-1* silencing significantly enhances Myogenin, Tnni2, troponin and α-actin expression, and it increases myotube formation in mouse myoblasts. Mechanistically, *Yam-1* can *cis*-regulate *miR-715* expression, which subsequently targets Wnt7b to impair the Wnt/β-catenin signaling. The *Yam-2* and *Yam-3* are located on mouse chromosome 3 and 15, respectively. *Yam-2* and *Yam-3* can enhance myogenic differentiation because *Yam-2* or *Yam-3* deficiency delays the myogenic program by reducing myogenic marker expression. In contrast, *Yam-4* silencing enhances myogenic differentiation. Overall, *Yam-1* and *Yam -4* inhibit myogenesis, while *Yam-2* and *Yam -3* promote myogenic differentiation. However, the molecular mechanisms by which these lncRNAs regulate myogenic differentiation remain to be further explored.

Metastasis-associated lung adenocarcinoma transcript 1 can regulate skeletal muscle differentiation (Schmidt et al., 2011; Watts et al., 2013) and its expression is highly upregulated in early regeneration stage of satellite cells, but downregulated when the fibers matured and regeneration completed (Chen X. et al., 2017). *Malat1* silencing accelerates muscle regeneration and enhances C2C12 differentiation, while *Malat1* over-expression has opposite effects. Further RNA-seq reveals that *Malat1* silencing up-regulates the expression of muscle-related genes, but down-regulates the expression of cell cycle-related terms, suggesting that Malat1 inhibits muscle differentiation. Mechanistically, *Malat1* suppresses myoblast differentiation as a novel downstream target of myostatin (Watts et al., 2013). Another study indicates that there is a regulatory axis of *miR-181a-Malat1*-MyoD/Suv39h1 during myogenesis because pro-myogenic miR-181a suppresses *Malat1* expression during differentiation (Chen X. et al., 2017). The reduced *Malat1* lncRNA is replaced by activating complex in the *MyoD* gene locus and subsequently induces MyoD expression and myoblast differentiation (Chen X. et al., 2017). There are other lncRNAs that participate in regulation of myogenesis. Collectively, there are several lncRNAs with diverse functions to regulate skeletal muscle myogenesis. Some of these lncRNAs contribute to the process of skeletal muscle dysfunction and may be potential therapeutic targets (Wang L. et al., 2015).

## LncRNA in Neurogenesis

Neural ESC differentiation is regulated by a complex network and understanding it may help with generating of high-quality neuronal stem cells for disease intervention. Transcriptome profiling analysis has identified many lncRNAs that are differentially expressed during neural differentiation. These indicate that lncRNA can regulate neural differentiation (Mercer et al., 2010).

A genome wide shRNA library targeting 1280 lncRNAs in mouse genome has identified nearly 20 lncRNAs that are essential for neuronal stem cell pluripotency (Lin et al., 2014). *TUNA* is a pluripotency-associated lncRNA that is exclusively expressed in the CNS. *TUNA* silencing in ESCs arrests neural differentiation. In mechanism, *TUNA* can interact and recruit multiprotein complex (PTBP1, hnRNP-K, and nucleolin) to their gene promoters to induce H3K4me, increasing Nanog, Sox2, and Fgf4 activity (Lin et al., 2014). *LncRNA-1604* is another lncRNA essential for neural differentiation of mESCs that is predominantly expressed in the brain and striatum. *LncRNA-1604* deficiency inhibits ectoderm differentiation and decreases the expression of neural progenitor cell markers (Sox1, Nestin, Zfp521, Pax6, and N-cad). Further luciferase and RIP assays demonstrate that *lncRNA-1604* can interact and sponge *miR-200c* to suppress the expression of the core transcription factors ZEB1/2 and the subsequently neural differentiation (Weng et al., 2018).

Maternally expressed gene 3 is derived from the DLK1-DIO3 imprinted locus. MEG3^–/–^ hESCs decreases the neural lineage differentiation rate by downregulating neural lineage marker expression, while induction of MEG3 expression has the opposite effect. Decreases in DLK1-DIO3-imprinted locus-induced lncRNAs can decrease neural lineage differentiation potential (Mo et al., 2015). *Evf2* is transcribed from the *Dlx5* and *Dlx6* intergenic region, and it plays a vital role in early hippocampal development. *Evf*2 deficiency reduces the number of GABAergic neurons, impairs synaptic inhibition in the hippocampus and dentate gyrus during early postnatal development (Feng et al., 2006). Mechanistically, the 5′ UCE region of *Evf2* can repress the short-range targets Dlx5/6 and the long-range targets Rbm28 and Akr1b8, while the 3′ UCE of *Evf2* can activate the long-range targets Umad1 and Lsm8. Thus, *Evf2* regulates neuronal differentiation through ncRNA-dependent topological and transcriptional control (Feng et al., 2006).

Recent microarray analyses reveal that 35 lncRNAs are up-regulated during neuronal differentiation of hESCs, including *RMST*, *DalI* and *PAUPAR* (Ng et al., 2012, 2013). *RMST* is specifically expressed in the brain and crucial for neurogenesis (Ng et al., 2012, 2013). *RMST* expression is regulated by two neural-specific transcription factors of PAX2 and REST. *RMST* can bind to SOX2 and is essential for SOX2 binding to the promoter regions of neurogenic transcription factors (Ng et al., 2012, 2013). While *DalI* can bind to crucial neurogenic transcription factor, POU3F3, and many of others involved in regulating cell cycling and neuronal development (Chalei et al., 2014). *DalI* deficiency inhibits neural differentiation in mouse N2A cells. Mechanistically, *DalI* can target gene expression and DNA methylation via interacting with DNMT1 (DNA methyltransferase) and POU3F3 (Ramos et al., 2016) while *PAUPAR* can target PAX6 expression (Vance et al., 2014). In contrast, lncRNA *Pnky* is expressed in human and mouse neural stem cells and its silencing enhances neuronal differentiation, indicating its inhibitory function (Keppetipola et al., 2016). *Pnky* can interact with PTBP 1 to regulate neuronal differentiation-associated gene transcription (Ramos et al., 2015; Keppetipola et al., 2016). Therefore, there are many lncRNAs that regulate neural differentiation of human and mouse ESCs positively and negatively by targeting various genes. It is important to further investigate how these *lncRNA*s regulate the dynamic neurogenesis process.

## LncRNA in Cardiovascular Development

Cardiovascular diseases are human health threats due to their high mortality and therapeutic strategy with CPCs has been attractive. However, CPC identification remains challenging. Therefore, it is imperative to understand specific cardiac lineage differentiation (Bruneau, 2013). Recently, emerging evidence uncovers that lncRNAs are crucial for cardiovascular development and maintaining cardiac integrity (Yang et al., 2014; Devaux et al., 2015).

Heart Brake LncRNA 1 is a human-specific lncRNA related to cardiomyocyte differentiation. *HBL1* over-expression suppresses cardiomyocyte differentiation by down-regulating GATA4, CTNT, NKX2.5, TBX5, TBX20 and α-MHC expression while its silencing has opposite effects (Liu et al., 2017). Mechanistically, *HBL1* can interact and sponge *miR-1*, a key cardiomyocyte differentiation promoter, to impair cardiomyocyte differentiation (Lu T.Y. et al., 2013; Liu et al., 2017). LncRNA *Braveheart* (*Bvht*) is exclusively expressed in the heart and can promote cardiovascular lineage commitment of mESCs by activating functional upstream genes of the MesP1, a permissive regulator of multipotent cardiovascular progenitor (Bondue et al., 2008, 2011). Further SHAPE probing and DMS probing assay demonstrate that *Bvht* secondary structure has a 5′ AGIL and a 11 nt motif in this 5′ AGIL, which is necessary for the cardiac differentiation process and cardiac transcription factor expression. Mechanistically, Bvht can antagonize the cardiac differentiation process negative regulator, cellular CNBP, the zinc finger TF (Xue et al., 2016) and interact with SUZ12 to epigenetically regulate cardiomyocyte differentiation. Hence, lncRNA may regulate gene expression though specific motifs.

*Fendrr* is a lateral mesoderm-specific and essential regulator for proper heart development in mice. *Fendrr* is located upstream of the 5′-end of *Foxf1*, and it is predominantly localized in the nucleus. Its mutants impair LPM lineages development, induces myocardial dysfunction and may be responsible for embryonic death. *Fendrr* deficiency down-regulates lateral plate or cardiac mesoderm differentiation-associated transcription factor expression, decreases H3K27 trimethylation, and/or increases in H3K4 trimethylation in the PRC2 occupied gene promoters. Mechanistically, *Fendrr* binds to the *Foxf1* and *Pitx2* gene promoters and represses the targeted gene expression via increasing PRC2 occupancy and H3K27me3 trimethylation (Grote and Herrmann, 2013; Cekin et al., 2018). In addition, *Fendrr* may regulate atherosclerosis development (Cekin et al., 2018). *CARMEN* is a human super enhancer-associated lncRNA with three isoforms that is highly expressed in differentiating CPCs to positively regulate cardiac differentiation. *CARMEN* deficiency impairs the capacity of human CPCs to differentiate into cardiomyocytes and significantly reduces the expression of cardiac transcription and differentiation makers (GATA4, NKX2.5, TBX5, and others). Therefore, *CARMEN* may initiate a cardiogenic differentiation program after damage, and it may be a potentially attractive therapeutic target for future regenerative and cell-based therapies (Bruneau, 2013).

A previous RNA-seq study has shown that lncRNAs, *TTN-AS1*, *ALIEN* and *PUNISHER* are differentially expressed in cardiovascular differentiation (Li Y. et al., 2017). *TTN-AS1* and the TANC1 are highly co-expressed during heart differentiation. *TTN-AS1* is regulated by MITF and TBX2, but its function during cardiovascular differentiation has not been explored (Li Y. et al., 2017). *ALIEN* is expressed in the nucleus and perinuclear regions of CPCs, and it can positively regulate cardiovascular development because *ALIEN* silencing upregulates the expression of genes involved in cell adhesion and extracellular matrix remodeling. However, it down-regulates angiogenesis and blood vessel development-associated gene expression. *PUNISHER* is an antisense transcript of the AGAP2 gene that is expressed in the cytoplasm of differentiated endothelial cells. *PUNISHER* expression is correlated positively with endothelial cell transcription factor (TAL1 and FOXC1) and vascular development related genes. PUNISHER deficiency downregulates histone H3 phosphorylation, and impairs human vessel maturation (Kurian et al., 2015).

Recently, Frank et al. (2019) defined a class of divergent lncRNAs in a hESC-based cardiac differentiation model. The so-called yin yang lncRNAs (yylncRNAs) exhibited the same expression pattern as their protein-coding counterparts. Among those lncRNAs, *yylncT* was identified as a mesodermal commitment specific lncRNA. It was transcribed from the mesoderm specifier BRACHYURY (T) locus and expressed in parallel with T during hESC cardiac commitment. Mechanically, *yylncT* worked as an essential activator of T by inhibiting DNMT3B activity to maintain the hypomethylation at the T/yylncT locus (Frank et al., 2019). Collectively, these lncRNAs act as stage-specific regulators of cardiovascular development. Technological development has discovered more and more functional lncRNAs in cardiovascular development. Further exploration will offer us more details about the stage specific regulation of lncRNAs in cardiomyocyte differentiation.

## LncRNA in Epidermopoiesis and Hematopoiesis

Anti-differentiation ncRNA is preferably expressed in progenitor keratinocytes, but significantly down-regulated in terminally differentiated cells (Kretz et al., 2012). *ANCR* deficiency reduces keratinocyte differentiation marker expression, including transcription factors that promote differentiation. Mechanistically, *ANCR* can recruit the PRC2 complex to the epidermal differentiation related *MAF* and *MAFB* promoters although ANCR can also promote osteoblast differentiation by up-regulating *Runx2* expression (Chen and Zhang, 2016). Transcriptome sequencing reveals that *TINCR* is one of the most highly induced lncRNAs in the cytoplasm of keratinocyte progenitors during keratinocyte differentiation. *TINCR* deficiency decreases differentiation-related gene expression and affects the formation of lipid barrier and keratohyalin granules in keratinocytes. PMA and RIA-seq demonstrate that *TINCR* can bind to mRNA decay protein STAU1, and interact with and stabilize MAF and MAFB mRNAs, as well as CALML5 protein to promote keratinocyte differentiation (Kretz et al., 2013; Lopez-Pajares, 2016). *HULC* can promote ADSCs differentiation into epithelial and smooth-muscle-like cells. Its overexpression increases the expression of Uroplakin-II, AE1/AE3, α-SMA, SM-MHC, Calponin, and SM-22α. Mechanistically, *HULC* positively regulates BMP9 expression and the Wnt/β-catenin activation, but it inactivates the Notch signaling during ADSC differentiation (Li et al., 2018).

Hematopoietic stem cells can differentiate into different types of blood cells. Besides transcription factors and miRNAs, recent studies reveal that lncRNA participates in hematopoiesis, particularly in the myeloid lineage development. The lncRNA *EGO* is crucial for an eosinophil development (Wagner et al., 2007). *EGO* silencing impairs the expression of basic protein and neurotoxin that are essential for eosinophil development, suggesting that *EGO* can positively regulate eosinophil lineage differentiation (Wagner et al., 2007). LncRNA also regulates the granulocyte and monocyte formation process. *HOTAIRM1* is a human HoxA cluster transcribed antisense lncRNA that is highly expressed in myeloid progenitor cells upon granulocytic differentiation. *HOTAIRM1* silencing inhibits HoxA1, HoxA4, CD11b and CD18 expression, impairing myeloid cell differentiation (Zhang et al., 2009). LncRNA *HoxBlinc* is a specific marker for Flk1 + mesoderm that can promote hematopoietic differentiation. Mesoderm-derived Flk1 + cells can be induced to be cardiogenic and hemangiogenic progenitors. HoxBlinc knockout downregulates the expression of *HoxB* gene and other cardiac/hematopoietic differentiation related genes and the components of the Notch signaling. In mechanism, *HoxBlinc* can bind to the HoxB genes and activated HoxB gene expression through recruiting the Setd1a/MLL1 complex and mediating long-range chromatin interactions. *HoxBlinc* can also regulate the Wnt/Notch signaling and Hox pathways (Deng et al., 2016).

## Conclusion and Further Perspective

As a major part of mammal genome, lncRNAs play essential roles in determining stem cell fates via different mechanisms. Some lncRNAs may be involved in different biological processes. For example, lncROR may maintain stem cell pluripotency (Wang et al., 2013) and promote the osteogenic differentiation process (Feng L. et al., 2018). *MEG3* is a positive regulator of both osteogenesis (Zhuang et al., 2015) and neurogenesis (Mo et al., 2015), while it acts as an adipogenesis inhibitor (Li Z. et al., 2017). LncRNA regulation presents a complex molecular interaction network. Some pluripotent reprograming regulatory lncRNAs have been recently identified using CRIST-seq (Zhang et al., 2019). They form a regulatory network around the *Oct4* and *Sox2* promoters and affect reprograming through epigenetic pathways, including coordination of intrachromosomal loops, alteration of the methylation levels, and activation of the eRNA pathway for stemness genes (Jia et al., 2020; Wang et al., 2020). It is interesting to explore whether these lncRNAs work in coordination, and what their spatial and temporal specialties are in regulating the fate of stem cells.

**FIGURE 3:**
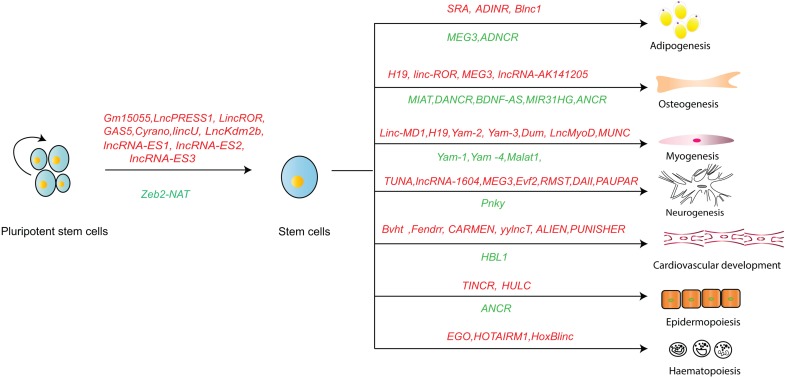
An overview of lncRNA in regulating stem cell fate. Letters in red: positive regulation; Letters in green: negative regulation.

The current understanding of lncRNA is more comprehensive due to improved technologies for lncRNA functionality, structure and interacting partners. LncRNA is important player in stem cell pluripotency maintenance, differentiation and their dysfunction-related human diseases through different mechanisms. The recently discovered lncRNAs that maintain stem cell pluripotency and determine their lineage differentiation are summarized in Figure 3. Interestingly, besides sequence conservation, the RNA structure and the location of lncRNAs seem to be good indicators for their function. Since lncRNA regulate somatic cell reprograming and stem cell differentiation, the manipulation of lncRNA may develop better protocols for efficient somatic cell reprograming and stem cell differentiation. There are new approaches to understand lncRNA regulation. For example, RAT-seq enables identification of the genome-wide chromatin binding sites for a specific lncRNA (Chen et al., 2018; Du et al., 2018). CRIST-seq could identify lncRNAs within the regulatory elements of stemness genes (Zhang et al., 2019). RIP, cross-linking and immunoprecipitation (CLIP), and RNA pull-down techniques are used for identifying the interaction proteomes of lncRNAs (Cao et al., 2019). These approaches will uncover more important roles and new mechanistic insights through which lncRNAs regulate biological process and relevant diseases. Conceivably, these new insights may also reveal new therapeutic targets and aid in design of new therapies for human diseases (Dey et al., 2014a).

## Author Contributions

JC, YW, and CW collected the data. JC wrote the manuscript. J-FH and WL provided guidelines and edited the manuscript. All authors read and approved the final manuscript.

## Conflict of Interest

The authors declare that the research was conducted in the absence of any commercial or financial relationships that could be construed as a potential conflict of interest.

## Abbreviations

ADNCR: adipocyte differentiation-associated long non-coding RNA
ADSC: adipose-derived stem cell
AGIL: asymmetric G-rich internal loop
ALP: alkaline phosphatase
ANCR: anti-differentiation ncRNA
CRIST-seq: chromatin RNA *in situ* reverse transcription sequencing
BMP: bone morphogenetic protein
BMSC: bone mesenchymal stem cell
CEBPA: CCAAT Enhancer Binding Protein α
CeRNA: competing endogenous RNA
ChIP-Seq: chromatin immunoprecipitation with the next-generation DNA sequencing
CNBP: nucleic acid binding protein
CNS: central nervous system
CPCs: cardiac precursor cells
DANCR: differentiation antagonizing non-protein coding RNA
DLK1-DIO3: delta-like homolog 1 gene and the type III iodothyronine deiodinase gene
DPPA2: developmental pluripotency-associated 2
DRR: distal regulatory region
Dum: *Dppa2* upstream binding muscle lncRNA
Dusp9: dual specificity phosphatase 9
ESC: embryonic stem cell
Fendrr: Fetal-lethal non-coding developmental regulatory RNA
FGF4: fibroblast growth factor 4
Gas5: growth-arrest-specific transcript 5
hASC: human adipose-derived stem cell
HBL1: Heart Brake lncRNA 1
HBMSC: human bone marrow-derived mesenchymal stem cell
hESC: human embryonic stem cell
HnRNP-K: heterogeneous nuclear ribonucleoprotein K
HOTAIR: *HOX* transcript antisense RNA
ICM: inner cell mass
IMP2: IGF2-mRNA-binding protein 2
iPSC: induced pluripotent stem cell
LIF: leukemia inhibitory factor
LncPRESS: lncRNA p53 regulated and ESC associated 1
LncRNA: long non-coding RNA
LPM: lateral plate mesoderm
Malat1: metastasis-associated lung adenocarcinoma transcript 1
MEG3: maternally expressed gene 3
mESC: mouse embryoid stem cell
MesP1: mesoderm posterior 1
MHC: myosin heavy chain
MIAT: myocardial infarction-associated transcript
MITF: microphthalmia-associated transcription factor
MLL: mixed-lineage leukemia
MUNC: MyoD upstream ncRNA
PAX2: paired box gene 2
PMA: protein microarrays
PPAR: peroxisome proliferator-activated receptor
PRC2: Polycomb Repressive Complex 2
pRNA: promoter associated RNA
PTBP: poly pyrimidine tract-binding protein
RAT-seq: RNA reverse transcription-associated trap sequencing
REST: neuron-restricting silencing factor
RIA-seq: RNA interactome sequencing
RIP: RNA immunoprecipitation
RMST: Rhabdomyosarcoma 2-associated transcript
RNA-Seq: RNA transcriptome sequencing
RNP: ribonucleoprotein
SRA: steroid receptor RNA activator
SRCAP: Snf2 related CREBBP activator protein
TANC1: tetratricopeptide repeat ankyrin repeat and coiled-coil containing 1
TBX2: T-box transcription factor 2
TINCR: terminal differentiation-induced ncRNA
TSS: transcription start sites
TUNA: Tcl1 upstream neuron-associated lncRNA
UCE: ultraconserved enhancer.
